# Reference data on hand grip and lower limb strength using the Nintendo Wii balance board: a cross-sectional study of 354 subjects from 20 to 99 years of age

**DOI:** 10.1186/s12891-019-2405-7

**Published:** 2019-01-12

**Authors:** F. Eika, A. W. Blomkvist, M. T. Rahbek, K. D. Eikhof, M. D. Hansen, M. Søndergaard, J. Ryg, S. Andersen, M. G. Jorgensen

**Affiliations:** 1 Department of Geriatric and Internal Medicine, Aalborg University Hospital, Hobrovej 18-22, 9000 Aalborg, Denmark; 2 Department of Geriatric Medicine, Odense University Hospital, Odense, Denmark; 3 Department of Health Science and Technology, Aalborg University, Aalborg, Denmark; 4 Institute of Clinical Research, University of Southern Denmark, Odense, Denmark; 5 Department of Clinical Medicine, Aalborg University, Aalborg, Denmark

**Keywords:** Nintendo Wii balance board, Isometric hand grip strength, Isometric lower limb strength, Muscle strength and aging, Normative data, Reference data, Force plate

## Abstract

**Background:**

Accurate assessment of isometric hand grip strength (HGS) and isometric lower limb strength (LS) are often limited to specialized clinics due to high costs and need for specialized equipment and personnel. A mobile and user-friendly device would facilitate a wider use of these measures in the clinical setting. The Nintendo Wii Balance Board (WBB) is a novel and pragmatic tool that has been validated for measuring muscle strength and other clinically relevant physiological variables. However, reference data for HGS and LS are lacking. The purpose of the current study is to establish reference data for HGS and LS in individuals ≥20 years of age using the WBB method, and to characterize the effects of age in these measurements.

**Method:**

Healthy participants were recruited at various locations and their HGS and LS were tested by six assessors using the WBB. Reference data were analysed and presented in age-groups, while the age-related change in HGS and LS was tested and characterized with linear regression models.

**Results:**

Three hundred and fifty-four participants between 20 and 99 years of age were tested. Data are presented separately according to gender and the following age categories: 20–29, 30–39, 40–49, 50–59, 60–69, 70–79, and 80+, and presented in absolute values as well as percentiles. The main findings were; (1) Significantly higher HGS and LS among males compared to females and for the dominant limb compared to the non-dominant limb, (2) a significant decline in strength with increasing age, and (3) the rate of decline increased significantly (i.e. it was non-linear) with age for HGS, but not for LS.

**Conclusion:**

This study reported reference data with percentiles for a novel method for assessing HGS and LS. Data were consistent with previously known effects of age and gender on HGS and LS. The presented data may supplement future trials using the WBB in research or in the clinical setting.

## Background

Normal musculoskeletal function, especially adequate strength in upper and lower extremities, is important for coping with everyday activities, and is a prerequisite for participating in sports, manual labour etc. [1]. Abnormal musculoskeletal function and low muscle strength has been associated with future disability [2], increased fall risk [3–8], and mortality [9–12], leading to significant healthcare expenditures [13]. The ability to accurately assess and monitor musculoskeletal function is important during growth, aging, training, rehabilitation, scientific research, and when screening for fall risk in older adults [14]. Muscle strength can be assessed either during a dynamic concentric/eccentric or isometric contraction. The most common method for assessing strength in the clinical setting is isometric manual muscle testing (subjective scale from 0 to 5). Simplicity, swiftness, and easy application makes it a popular method in everyday clinical practice. However, its crudeness [15] makes it unreliable for strength assessment both within and between different assessors [16]. Contrary to this are hand grip- and stationary isometric/isokinetic dynamometers, which are considered the reference standards for measuring hand grip strength (HGS) and lower limb strength (LS). The hand grip dynamometers have shown high test-retest reliability and concurrent validity [17–19]. Most widely used is the Jamar dynamometer (JD), but the apparatus is primarily found in specialized clinics as it provides data for a single clinical parameter (HGS) and comes at a fairly high price of 200 to 1200 USD [20].

In contrast to HGS assessment, the stationary isometric/isokinetic dynamometer for LS assessment has never gained ground for clinical use, mainly because of the need for large scale and expensive equipment. Hand-held-dynamometry has been a feasible alternative for measuring LS owing to its light weight, versatility, and relatively low price [21, 22]. However, the reproducibility of hand-held-dynamometry varies greatly depending on study population, operator, and muscle group tested [23]. In fact, the participant may overpower the tester [24] or the tester may overpower the participant [25]. In short, HGS and LS are useful parameters for detecting and monitoring individuals at high risk for adverse outcomes [12, 26] but the assessment using validated methods are usually restricted to universities and university hospitals. A promising new method for measuring HGS and LS is the use of a standard Nintendo Wii Balance Board (WBB) and customized software [20, 23, 27]. The method has a good validity when compared against the reference standards for HGS and LS [20, 23, 27]. It has also been shown to be valid for testing reaction time [28, 29], muscle force steadiness [30], and postural balance in younger [31] and older adults [32, 33]. Hence, the WBB has the potential to provide accurate assessment of several physiological measures, previously unavailable in a community setting. Still, reference data on HGS and LS using this method is lacking. Therefore, the aim of this study was to (1) establish reference data for HGS and LS in healthy individuals ≥20 years of age using the WBB method and (2) to describe the effects of age in these measurements.

## Method

### Study design & population

This was a cross-sectional study where all measurements (physiological and anthropometric) were collected during a single test session. Participants were recruited during the spring and summer of 2016 at various locations (e.g. university campus, malls, hospital staff, and senior citizen clubs) in Denmark. People were eligible for inclusion if they were ≥ 20 years of age and considered themselves at good health. Exclusion criteria were obvious cognitive problems (i.e. could not name present year or capital of Denmark), insufficient ability to stand unsupported for 30 s, neuromuscular deficits (e.g. parkinson, myastenia gravis, sequela after stroke, or severe polyneuropathy), or musculoskeletal disease (e.g fracture or orthopedic surgery within the last 6 months, alloplasty in the last 2 years, muscular dystrophy, or polymyositis rheumatica). Participant characteristics included age, gender, weight, height, hand and leg dominance, smoking status, and number of prescribed drugs used daily. Finally, data was collected for the participants’ physical activity at work and during leisure time, using a method similar to the Copenhagen City Heart Study [34].

### Equipment and software

The WBB (Nintendo, Kyoto, Japan) is a small force plate, instrumented with four uni-axial stain gauge transducers positioned in each corner, similar to what is typically seen in professional force platforms. Data is transferred wirelessly to a personal computer via Bluetooth Human Interface Device and onto the FysioMeter® software (Bronderslev, Denmark). From each of the transducers, channels of 16-bit digital data at approximately 100 Hz are filtered using a 4th order Butterworth filter (cut-off 20 Hz).

### Overall experimental procedure

Six operators measured balance, reaction time, HGS, and LS. The operators were three medical doctors (FE, AWB, and MTR), two nurses (KDE and MS), and one physiotherapist (MDH). Testing procedures were standardized and operators were synchronized with each other at the Department of Geriatrics, Aalborg University Hospital, Denmark prior to data collection. To minimize systematic bias, each operator collected approximately seven to nine individuals from each of the seven age groups (20–29, 30–39, 40–49, 50–59, 60–69, 70–79, and 80+ years).

After explaining the testing procedures, acquiring oral consent, and collecting anthropometric data, the measurements were performed in the following order: balance, reaction time, HGS, and LS.

This order was chosen as previous studies have shown that testing maximum muscle strength before postural balance could affect the balance results [35]. Coefficient of variance were calculated during HGS and LS measurements. A coefficient of variance of more than 10% in consecutive measurements initiated a re-test of the lowest value to avoid excessive variance between the measurements. If the subsequent measurement was still more than 10% off the higher value, the higher value was re-tested.

Detailed protocols for assessment of balance and reaction time are available elsewhere [28, 32], and reference data on reaction time can be found in a separate publication [29].

### Hand grip strength (HGS) procedure

**Fig. 1:**
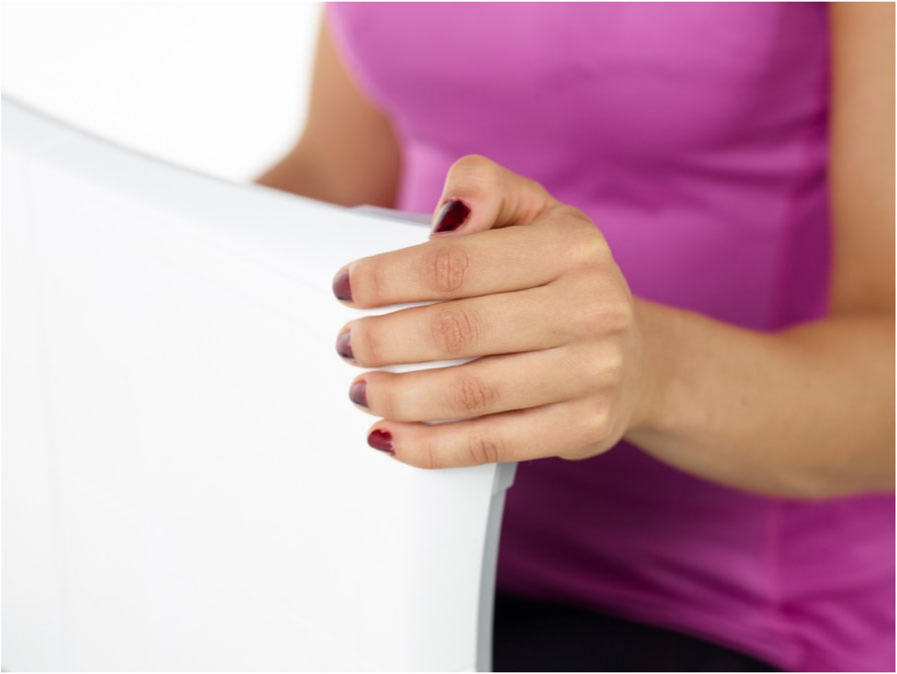
Participant squeezing one of the force transducers of the Nintendo Wii Balance Board during isometric hand grip strength testing

The participant was placed in a chair with the back straight and shoulders in their anatomical position. The WBB was held vertically, resting on the thighs, with the back of the board facing their torso at about 20 cm distance [20]. While squeezing the corner of the WBB with either left or right hand (Fig. 1), the participant and operator viewed the force-time curve in real time on the computer screen. After 2–3 submaximal recordings for familiarization, maximal isometric HGS was assessed beginning with the left hand and followed by the opposite hand in an alternating fashion for a total of two recordings per side. The participant was encouraged to squeeze as long and hard as possible until a plateau on the force-time curve was reached. Then the operator told the participant to stop squeezing. If such a plateau was not reached, the participant was instructed to stop when he or she was unable to further increase the force recording on the force-time curve. The output used for further analyses for each side was the average of the two maximal isometric HGS recordings.

### Lower limb strength (LS) procedure

**Fig. 2:**
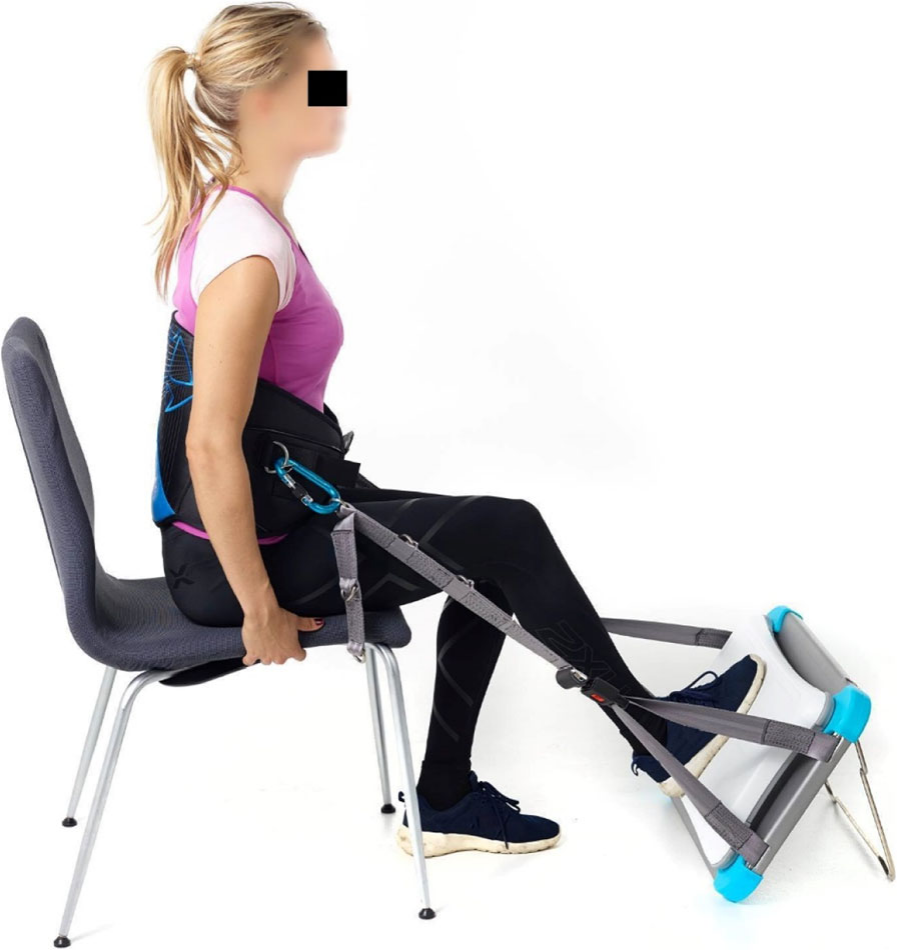
Nintendo Wii Balance Board mounted to the aluminium frame and attached to the harness using straps, during isometric lower limb strength assessment

The WBB was mounted to a custom-made aluminium frame and attached via belts to a harness around the participants hips prior to testing (Fig. 2). With the participant placed in a standard chair (seat height approximately 45 cm) with the back straight and shoulders in their anatomical position. The equipment was adjusted so that the knee was flexed at 60 degrees during loading. Grabbing onto the sides of the chair, one leg was placed in the middle of the WBB, before trials of 2–3 submaximal loads and two maximal loads were performed. Submaximal loads were performed for habituation, to reduce variance and to ensure correct knee angle while pressing. Four tests were performed (two for each leg), in alternating fashion, starting with the left leg. A force-time curve provided real time visual feedback to the participant during testing, as this has shown to influence output [36]. For the two maximal recordings, the participant was encouraged to squeeze as long and hard as possible until a plateau was reached on the force-time curve. As with HGS, if such a plateau was not reached, the participant was instructed to stop when he or she was unable to further increase the force recording on the force-time curve. The output used for further analyses for each side was the average of the two maximal isometric LS recordings.

### Statistical analysis

All data were plotted in a Microsoft Excel spreadsheet and statistical analyses were performed using the Statistical Program for the Social Sciences (SPSS, version 24). All results are given in mean ± standard deviation (SD) for normally distributed data (Shapiro-Wilk) and median with interquartile range for non-normal distributions. From the FysioMeter® software, four variables of muscle strength were extracted for each participant: HGS for dominant and non-dominant side (HGS-D, HGS-ND, respectively) and LS for dominant and non-dominant side (LS-D, LS-ND, respectively). Individuals were divided according to age groups (20–29, 30–39, 40–49, 50–59, 60–69, 70–79, and 80+ years) and gender (male, female). Using outlier labelling rule [37], outliers were identified and subsequently winsorized, except for extreme values, where measurement errors was suspected and hence, removed. For each gender and age-group, the 10, 25, 75, and 90 percentiles for the four strength variables were extracted. One-sample t-test and independent t-test was used to test for difference between the mean values for each side (dominant and non-dominant) and gender, respectively. Non-normal distributed data were tested with non-parametric tests, i.e. Mann-Whitney U test and Wilcoxon Signed-Rank test.

To investigate the age-related changes in HGS and LS, four linear regression models were calculated using the mean values for hand and feet for each gender. Assumptions of linearity and homoscedasticity were assessed with the standardized residuals plotted against predicted values, while the assumption of normal distributed errors and autocorrelations were assessed with the histogram of residuals and Durbin-Watson test (accepted value between 1.5 and 2.5), respectively [38]. Violations of linearity or homoscedasticity was corrected by transformations. The presence of non-linear relationships were statistically tested with hierarchical multiple regression using a quadratic model (i.e. adding the age squared as an independent variable to the linear regression models).

### Ethics

Participants gave oral consent and the study was approved by the ethics committee of the North Jutland Region, Denmark.

## Results

A total of 354 men and women, between 20 and 99 years of age, were tested. Participant characteristics are shown in Table 1. Absolute values (kg) and percentiles (10, 25, 75, and 90%) of HGS and LS for each group are shown in Tables 2 and 3, respectively. Out of 1416 observations, nine outliers were detected, of which eight were winsorized [37] and one was omitted.

**Table 1:** Study-population characteristics

Age group (years)	Gender & number	Age (years)	Height (cm)	Weight (kg)	BMI (kg/m^2^)	Medicine (number)	Smoking (N;C + P) %	Physical activity level work	Physical activity level leisure
20–29	M 22	24.5 ± 2.5	184 ± 4.8	85 ± 11.7	23.2 ± 3.2	0 [0–1]	86;14	2;1	3;1.25
	F 36	25;3.8^a^	167 ± 5.8	62 ± 7.5	23.2 ± 3.2	0 [0–1]	86;14	2;1	3;1
30–39	M 15	33.3 ± 2.4	182 ± 4.8	85 ± 17.8	25.9 ± 5.7	0 [0–0]	73;27	2;0	3;1
	F 30	33.5;6.3^a^	167 ± 5.9	73 ± 16.3	26.0 ± 5.7	0 [0–1]	80;20	2;1	2.5;1
40–49	M 20	44.7 ± 2.9	182 ± 6.0	90 ± 15.4	27.2 ± 4.7	0 [0–0]	55;45	2;1	3;2
	F 21	46;5.0^a^	170 ± 4.5	79 ± 15.3	27.2 ± 4.7	0 [0–0]	67;33	2,5;1,75	3;1.5
50–59	M 16	54.9 ± 3.3	183 ± 6.1	87 ± 12.4	25.9 ± 3.8	0 [0–1]	63;38	2,5;1	3;1
	F 30	54.4 ± 2.9	166 ± 6.9	73 ± 12.9	25.8 ± 3.9	1 [0–2]	53;47	2;2	2;1
60–69	M 19	65.3 ± 2.3	180 ± 7.0	94 ± 18.8	27.5 ± 5.7	0 [0–4]	42;58	2;2	3;1
	F 35	66;6.0^a^	166 ± 5.6	73 ± 14.2	27.6 ± 5.7	1 [0–2]	60;40	3;1	3;1
70–79	M 32	73.5 ± 2.8	178 ± 6.2	86 ± 10.9	26.9 ± 3.9	1 [0–3]	44;56	^b^	3;1
	F 33	73.6 ± 2.8	167 ± 5.4	74 ± 12.7	26.9 ± 3.9	1 [1–5]	64;36	^b^	2;1
80+	M 20	85.6 ± 4.1	175 ± 5.1	82 ± 10.6	25.8 ± 3.5	3 [0–6]	35;65	^b^	2;2
	F 25	85.6 ± 4.0	163 ± 6.6	67 ± 11.6	25.7 ± 3.6	3 [1–5]	52;48	^b^	3;1.5

Anthropometric data for the different age and gender groups. Medicine refers to number of drugs used daily. Smoking is divided into never (N) and current (C) or prior (P) and given in percentages. Physical activity at work and during leisure time is reported in medians from 1 (least active) to 4 (most active) and variance as interquartile range

^a^median (interquartile range)

^b^An insignificant number of participants were working (i.e. most participants where fully retired)

**Table 2:** HGS and LS (absolute values)

Age group(years)	Gender & number	HGS-D	HGS-ND	LS-D	LS-ND
20–29	M 22	35.4 ± 8.4	33.0 ± 7.0	238.8 ± 56.5	232.5 ± 58.1
	F 36	21.7 ± 3.5	19.9 ± 4.5	172.8 ± 37.5	165.1 ± 38.0
30–39	M 15	37.5 ± 6.9	35.3 ± 4.2	250.7 ± 46.6	241.6 ± 42.6
	F 30	22.8 ± 4.1	20.8 ± 3.6	152.4 ± 40.0	149.2 ± 37.4
40–49	M 20	37.4 ± 7.3	34.9 ± 8.1	206.8 ± 65.8	210.8 ± 64.9
	F 21	23.2 ± 3.9	21.3 ± 3.2	162.7 ± 52.9	158.5 ± 51.5
50–59	M 16	32.7 ± 5.3	31.2 ± 7.0	197.3 ± 46.4	181.9 ± 48.7
	F 30	20.8 ± 4.2	18.7 ± 4.5	128.9 ± 46.7	125 ± 42
60–69	M 19	30.3 ± 7.1	28.1 ± 7.3	174.7 ± 57.0	168.6 ± 50.5
	F 35	17.9 ± 2.9	15.7 ± 3.2	104.3 ± 31.4	100.7 ± 30.6
70–79	M 32	25.0 ± 7.3	24.3 ± 6.4	148.2 ± 48.8	145.0 ± 46.6
	F 33	15.7 ± 3.7	14.4 ± 3.0	98.9 ± 34.9	99.1 ± 35.7
80+	M 20	18.5 ± 4.6	17.6 ± 4.2	111.8 ± 36.4	108.7 ± 40.8
	F 25	11.9 ± 2.0	11.7 ± 2.7	53.5 (33.1)^a^	63.8 ± 28.3

Results from strength assessment for male (M) and female (F) in different age groups. Hand grip strength dominant (HGS-D), hand grip strength non-dominant (HGS-ND), isometric lower limb strength dominant (LS-D) and isometric lower limb strength non-dominant (LS-ND) are given in kilograms

^a^Mean 59.9 ± 27.9

The differences between male and female gender for HGS and LS values are all statistically significant with *p* < 0.001. Exceptions are; 40–49: LS-D (*p* = 0.012), LS-ND (*p* = 0.005). 50–59: LS-ND (*p* = 0.002)

**Table 3:** HGS and LS (percentiles)

Age group (years)	Gender & number	HGS-D 10,25,75,90	HGS-ND 10,25,75,90	LS-D 10,25,75,90	LS-ND 10,25,75,90
20–29	M 22	24,31,41,47	25,29,38,42	151,189,285,311	137,197,270,313
	F 36	17,20,24,26	14,17,23,25	116,149,202,223	108,134,194,218
30–39	M 15	27,33,44,46	28,33,37,41	176,209,297,310	184,206,277,303
	F 30	19,21,26,29	17,19,23,25	86,122,182,198	91,122,180,202
40–49	M 20	28,32,42,48	24,28,40,42	110,155,248,301	132,154,265,306
	F 21	18,20,26,29	16,19,24,25	91,114,204,226	85,111,199,223
50–59	M 16	27,29,35,42	23,27,35,42	135,158,244,264	101,153,216,239
	F 30	16,18,24,28	13,15,22,24	68,89,150,193	71,87,150,201
60–69	M 19	22,25,35,44	19,22,35,39	93,122,211,234	95,118,214,238
	F 35	14,16,20,22	12,13,18,20	62,84,120,156	62,80,120,158
70–79	M 32	16,19,31,36	16,19,30,34	94,111,175,217	92,105,179,205
	F 33	10,14,18,21	10,12,16,19	56,71,110,159	54,70,120,166
80+	M 20	10,16,21,25	11,15,21,23	65,79,145,164	56,73,145,174
	F 25	8.7,11,13,14	8.5, 9.7, 13, 16	31,40,73,110	32,43,77,111

10, 25, 75 and 90% - percentiles in kilograms for male and female in the different age groups

**Fig. 3:**
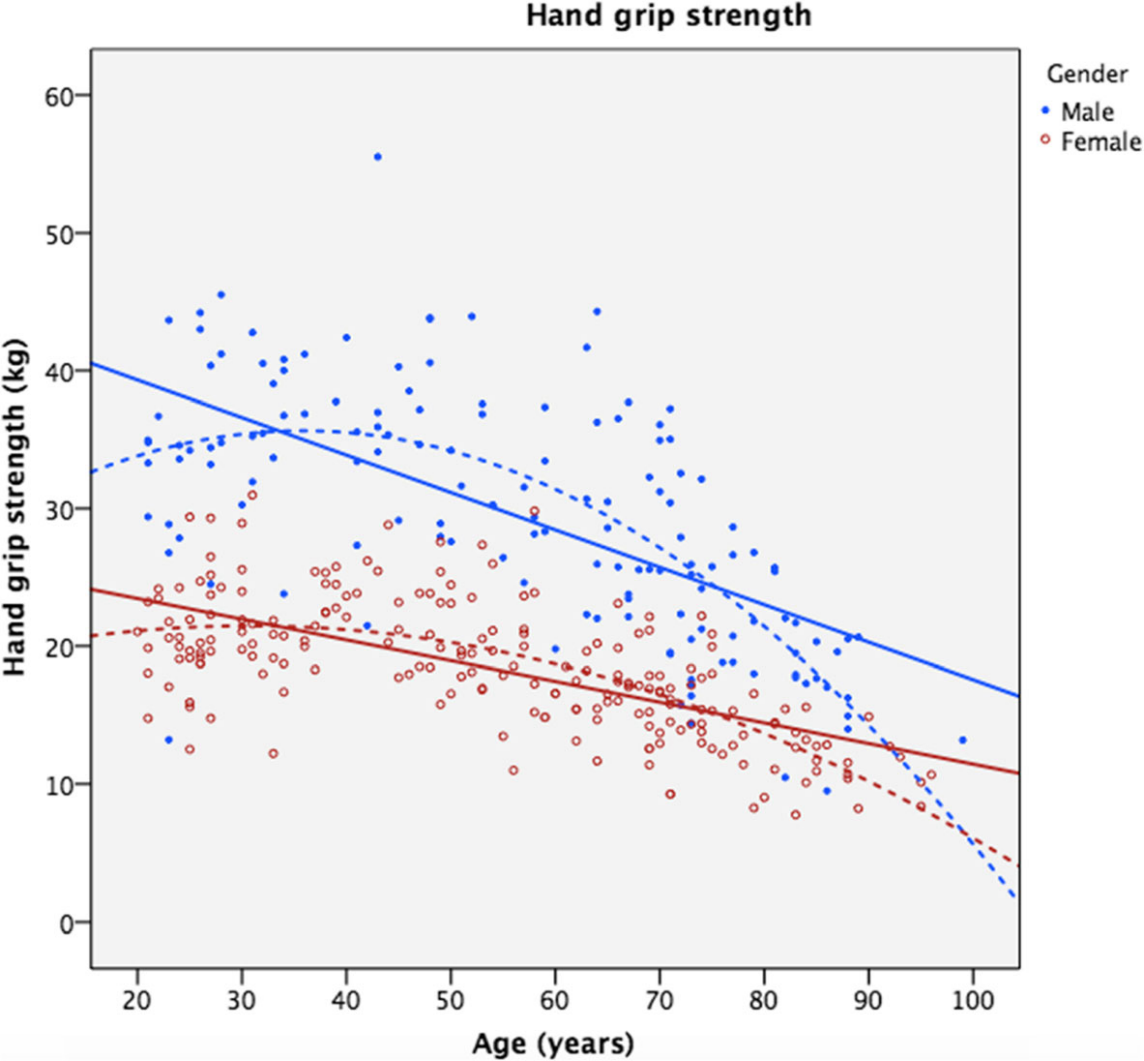
Hand grip strength data in kilograms as a function of age. Solid lines represent linear regression models, while dotted lines are quadratic regression models

**Fig. 4:**
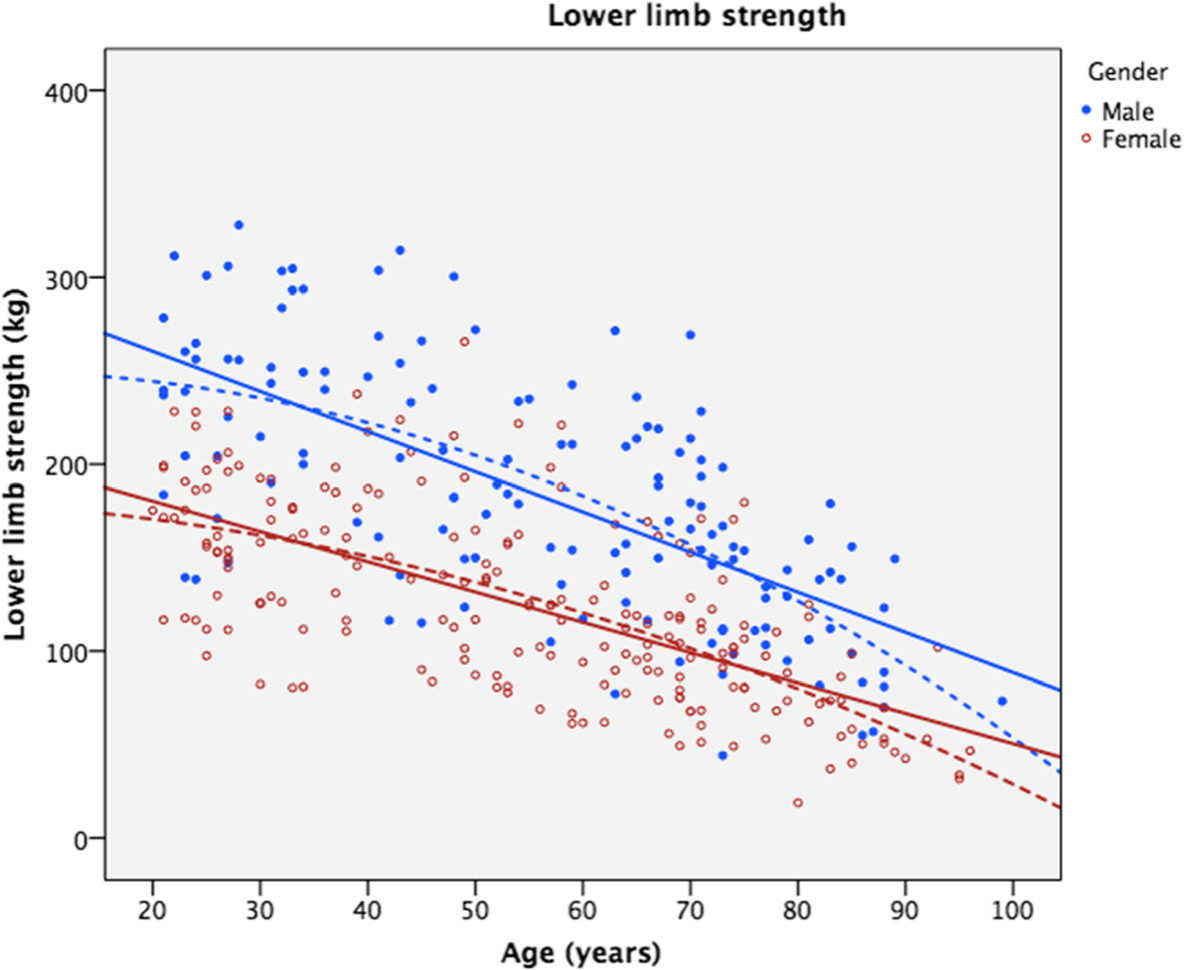
Lower limb strength data in kilograms as a function of age. Solid lines represent linear regression models, while dotted lines are quadratic regression models

As shown numerically (Table 2) and graphically (Figs. 3 and 4), both HGS and LS decreased with increased age in both genders. The linear regression (Table 4) showed a significant inverse relationship between age and strength. Age accounted for an average of 42 and 45% of the variation in HGS and LS, respectively. For HGS, the quadratic models gave a significant R^2^ change of 9.8% (F change = 28.4, *p* < 0.001) and 6.8% (F change 27.6, *p* < 0.001) for males and females, respectively. For LS, the quadratic models gave a statistically non-significant R^2^ change of 1.5% (F change = 3.9, *p* = 0.050) and 1.0% (F change = 3.7, *p* = 0.055), for males and females, respectively. These models are illustrated in Figs. 3 and 4 together with the raw data.

**Table 4:** Linear regression models for muscle strength and age

Variable	R	R squared	F-value (*p*-value)	Regression coefficient
Hand grip strength				
Male	.644	.414	100.5 (< 0.001)	−0.27 (95% CI: −0.33;-0.22)
Female	.648	.420	150.4 (< 0.001)	−0.15 (95% CI: −0.17;-0.13)
Lower limb strength				
Male	.674	.455	116.7 (< 0.001)	−2.14 (95% CI: −2.53;-1.75)
Female	.662	.436	159.3 (< 0.001)	−1.62 (95% CI: −1.87;-1.36)

There was a statistically significant gender difference (*p* < 0.001) with males being, on average, 11.1 kg (95% CI: 9.5; 12.7) and 57.9 kg (95% CI: 44.9; 70.8) stronger than females for HGS and LS, respectively. The dominant side was, on average, 1.7 kg (95% CI: 1.4; 2.0) and 3.8 kg (95% CI: 1.8; 5.8) stronger than the non-dominant side (*p* < 0.001) for HGS and LS, respectively.

## Discussion

This study reported reference data in males and females on HGS and LS using a standard WBB and investigated its relationship with age. The main findings were: (1) Significantly higher HGS and LS among males compared to females and for the dominant limb compared to the non-dominant limb, (2) a significant decline in strength with increasing age, and (3) the rate of decline increased significantly (i.e. it was non-linear) with age for HGS, but not for LS.

### Hand grip strength

Hogrel et al. reported HGS using JD and MyoGrip dynamometer in a population similar to that of this study [39]. For participants aged 20–80, the averaged HGS for males and females using the JD were 40.8 and 38.5 kg for the right and left side, respectively. Assuming right hand dominance, the corresponding values were 26.7 and 24.8 kg in our study sample, revealing a difference of 14.1 (35%) and 13.7 (36%) kg. This difference is consistent with the HGS reproducibility study, which found a systematic difference between the JD and WBB of 15.4 and 11.9 kg for the right and left side, respectively [20]. This systematic difference is also consistent with studies comparing JD and other dynamometers [39–42]. Also, supporting the external validity of the method, the mean HGS for the 30 subjects (both genders, mean age 69 years) tested in the WBB reproducibility study was 21.4 kg, while it was 21.5 kg for the participants between 60 and 79 years in the current study.

It was found that the dominant hand was stronger for every age group (on average 1.7 kg stronger) and males were generally stronger than females (on average 11.1 kg). These findings are consistent with other studies using different methods [39–41, 43], although the difference between dominant and non-dominant is sometimes small and statistically non-significant [44]. Moreover, the gender difference varied with age and with smaller difference for older adults compared to midlife and young adults. One reason could be a gender difference in survival, in which a larger proportion of surviving females are strong compared to surviving males. Another reason could be gender differences in the contribution of physical activity to maximal strength among young and midlife adults that evens out in older adults. Among younger individuals, the relative gender difference in age group 20–40 years of age was 40%. This is very similar to the reported relative gender difference in HGS for the same age group of using JD (42%) and Dynex-dynamometer (43%) [40].

The present study is also consistent with the expected inverse relationship between HGS and age due to decreasing neuromuscular function [39, 45, 46]. Similar to our findings, a large epidemiological meta-analysis using multiple dynamometers found an increase in HGS into adult life, peaking around 30 to 40 years of age, broad maintenance through midlife to around 60 years of age before declining [47]. Many factors may contribute to this late in life loss, such as decreased muscle protein synthesis, changes in body composition due to metabolic disorders, and reduced ability to exercise [46]. As found in other studies as well, this relationship is significantly curvilinear, i.e. the rate of decline increases with age [43, 44]. Our data do not point to reasons for this accelerating decline, but this is a topic of interest for future follow-up.

### Lower limb strength

Contrary to HGS, the absolute LS values reported in this study are not fully comparable to findings from the reproducibility study [23]. In a mixed group of 30 older adults (mean age 69), LS was on average 102 kg and 100 kg for dominant and non-dominant limb, respectively [23]. Combining the 60–69 and 70–79 age group from the current study, the averaged LS was 131 kg and 128 kg, respectively. Differences between study populations may explain some of this discrepancy. The former study included a relative higher proportion of women (70% vs 57% in this study) and participants had a smaller body size (on average 68 kg vs 74 kg for females, 83 kg vs 90 kg for males), suggestive of a lower muscle mass in that study. Although the ratio between strength and body mass could be used, absolute values are typically reported in the literature, probably because the relationship between body mass and strength is complicated by differences in body composition. The substantial gender difference in LS is consistent with this. Clearly, poor external validity of the method may also account for a part of the discrepancy observed. Consistent with this is the fact that the WBB method has higher reliability with HGS assessment [20, 23, 27], and therefore showed better external validity for the HGS assessment than with the LS assessment. Lastly, both the current study and the reproducibility study found a small, albeit statistically significant for a sufficiently large sample, difference in LS between the dominant and non-dominant sides.

Previous studies on LS most often report on isolated knee or hip movements (e.g. flexion of the knee or extension of the hip) and/or provide results in newton-meters [22, 45, 48], which are not directly comparable to our method or measurement. Still, we may compare the influence of gender and age reported in the literature, and this is consistent with our findings. On average, males are stronger and lower limb strength decreases with age [45, 48]. In more detail, both Danneskiold et al. [45] and Harbo et al. [48] reported a consistent linear decrease in isometric knee extension with increasing age for both males and females. However, statistical test for a non-linear relationship are missing in these reports [45, 48]. Furthermore, age accounted for more of the variation in LS than upper limb strength (i.e. higher *R*^2^ for LS) in their reports as well as in our regression models [45].

### Strengths and weaknesses with the study and method

In relative values the reference data obtained using WBB in the current study appear externally valid when compared to other larger studies on HGS and LS, particularly the effects of gender, age, and dominance on strength. In absolute terms, HGS data revealed the same systematic difference with the JD as found previously with the WBB and other dynamometers. For LS, there was a discrepancy when compared with the previous study using the WBB and very few studies have measured LS in a way that is comparable to this study. A strength of the current study is the relatively large sample tested with a rigid protocol, developed in a pilot study, to minimize systematic bias. When the protocol was in place, a 3 h test session was held with the six raters in order to synchronize the experimental procedures. Also, to minimize bias from a single rater on a particular age group, all raters collected data from every age group. A further strength is that the HGS and LS data were collected in the participants’ own home, or in a public location, as a battery of tests with balance and reaction time testing in order to simulate a community-visit risk assessment. However, the non-random selection of participants, the different testing environment (both location and time of day), and age groups compared to the validation studies are weaknesses that may introduce bias and hamper the generalizability of the results. Still, most of these limitations provide random noise to the data and the impact was reduced by the relatively large number of participants. A smaller systematic bias can be found in the age group 30–39 and 70–79, which has a relatively high number of individuals in the lower end of the age group. However, the results from these age groups follow the overall trend for all measures and the potential bias introduced is likely to be minimal.

A technical limitation with the method is that the WBB system lacks the ability to record horizontal shear forces. This primarily renders the system applicable to static or semi static testing conditions only, which is important to consider when evaluating a patient, as static and dynamic (eccentric/concentric) muscle strength may not follow the same pattern for all ages and conditions. As noted previously, the systematic difference in HGS between WBB or HDD and the reference standard (JD) hinder direct comparison of data [20]. A similar limitation exists in LS where the method also correlates strongly with the reference standard, but gives systematically lower absolute values [23, 27]. Interestingly, for bilateral LS, the WBB method has shown a higher intraclass correlation and a lower standard error of measurement and limits of agreement compared to the reference standard [27]. Future research may further elucidate advantages and disadvantages inherent in the different methods available. However, the LS assessment sometimes required that the rater stabilized the custom steel plate during maximal pressing. Also, the knee angle may have deviated from the approximate 60 degrees flexed position during pressing, which required repositioning of the straps attached to the harness. For more specific, albeit minor, limitations on each measure, these have been reported in the prior reproducibility studies [20, 27, 28, 32]. More importantly, the WBB is a widely available, inexpensive, versatile, and portable instrument, that has been validated for measuring multiple clinically relevant parameters. In the current study, multiple raters collected data in a home setting, demonstrating the practicality of the method. On average, a whole test session took about 35 min. It is a major strength that data on postural balance, reaction time, HGS, and LS can be collected within this short timeframe. The WBB appears to be gaining ground as a new and innovative measurement and exercise tool and the HGS and LS reference data established in this study could supplement further trials and clinical use.

## Conclusion

In this study, we reported reference data on a new objective and portable method for reliable HGS and LS testing in a healthy population of 354 participants in the ages between 20 and 99. The results showed decreased HGS and LS with age, lower HGS and LS in women compared to men, and a significant accelerated decline in HGS with age, which is in accordance to epidemiological studies on strength. The reference data can be used for rehabilitation purposes or future screening programs attempting to identify individuals at risk for fall accidents, frailty, and sarcopenia.

## Abbreviations

HGS: Isometric hand grip strength or hand grip strength
HGS-D: Dominant side
HGS-ND: Non-dominant side
LS: Isometric lower limb strength or lower limb strength
LS-D: Dominant side
LS-ND: Non-dominant side
WBB: Nintendo Wii Balance Board
JD: Jamar dynamometer
SD: Standard deviation
